# Sexual behaviour and STIs among MSM living with HIV in the PrEP era: the French ANRS PRIMO cohort study

**DOI:** 10.1002/jia2.26226

**Published:** 2024-03-10

**Authors:** Virginie Baltes, Paul de Boissieu, Karen Champenois, Louise Luan, Rémonie Seng, Asma Essat, Sophie Novelli, Bruno Spire, Jean‐Michel Molina, Cécile Goujard, Laurence Meyer, for the ANRS PRIMO cohort study group

**Affiliations:** ^1^ Inserm, CESP U1018 Le Kremlin‐Bicêtre France; ^2^ Université Paris Saclay Faculté de médecine Le Kremlin‐Bicêtre France; ^3^ AP‐HP Epidémiologie et Santé publique Hôpital Bicêtre Le Kremlin‐Bicêtre France; ^4^ Université de Paris Cité et Université Sorbonne Paris Nord Inserm, IAME Paris France; ^5^ Aix Marseille Univ Inserm, IRD, SESSTIM, ISSPAM Marseille France; ^6^ AP‐HP Service de maladies infectieuses Hôpital Saint‐Louis Paris France; ^7^ Université Paris Cité Paris France; ^8^ AP‐HP Service de médecine interne Hôpital Bicêtre Le Kremlin Bicêtre France

**Keywords:** MSM, sexual behaviours, PrEP, TasP, sexually transmitted infections/diseases, cohort studies

## Abstract

**Introduction:**

In a context of declining condom use and high sexually transmitted infection (STI) incidence, the diffusion of “treatment as prevention” (Tasp) and more recently pre‐exposure prophylaxis (PrEP) may have changed the sexual behaviour of newly diagnosed men who have sex with men (MSM) with HIV.

**Methods:**

Six hundred and nine MSM were enrolled and followed annually between 2014 and 2021 in the ANRS PRIMO Cohort (ClinicalTrials.gov:NCT03148964) from the time of HIV seroconversion. We studied changes over calendar time in sexual behaviour before and after HIV diagnosis. Factors associated with inconsistent condom use (ICU) after HIV diagnosis, PrEP use by partner(s) and bacterial STI acquisition were studied in random‐effects models.

**Results:**

In the 6 months preceding HIV diagnosis, the number of sexual partners decreased from a median of 10 (IQR: 4−19) in 2014 to 6 (3−11) in 2021. After HIV diagnosis, ICU increased from 57.1% (16/28) of visits in 2014 up to 84.2% (229/272) in 2020−2021. Up to 25% (63/229) of MSM with HIV in recent years reported the use of PrEP by their partner(s) as the reason for ICU; these MSM were less frequently in a stable relationship, had a higher number of sexual partners and higher education level than those who did not report the use of PrEP by their partner(s). STI incidence after HIV diagnosis increased between 2014 and 2016 and remained high afterwards. STI risk was no longer associated with PrEP use by partners after adjustment for the number of partners and calendar period. ICU, age below 35 years, not being in a stable relationship, higher number of sexual partners were independently associated with an increased risk of STI.

**Conclusions:**

Implementation of TasP and more recently PrEP has led to major changes in the sexual behaviour of MSM with HIV. ICU has become overwhelmingly prevalent, PrEP use by the partner increasingly being the reported reason for ICU, behind TasP, which remains the main reason. Characteristics of MSM at the time of diagnosis of HIV have changed, with fewer number of sexual partners today than in 2014, which must lead to broaden the indications for PrEP prescription. STIs incidence remains high in MSM with HIV and requires improvements in screening and prevention methods such as pre‐ or post‐exposition antibiotics or vaccines.

## 1 INTRODUCTION

Men who have sex with men (MSM) are particularly affected by the HIV epidemic worldwide [1, 2]. However, the number of new HIV diagnoses among MSM has declined in recent years in many high‐income countries [3, 4, 5, 6]. Several factors may have contributed to this. The development of powerful antiretroviral treatment allowed the “treatment as prevention” strategy (TasP) from the late 2000s for people with HIV [7, 8, 9, 10]. We previously described how sexual activity and condomless sex have increased in MSM living with HIV with the diffusion of TasP [11, 12]. More recently, pre‐exposure prophylaxis (PrEP) with emtricitabine/tenofovir has been increasingly prescribed to HIV‐negative MSM. When adherence is optimal, PrEP has been proved to be highly effective in reducing HIV incidence among MSM [13, 14, 15]. In France, PrEP has notably been indicated since 2016 for MSM or transgender individuals reporting multiple partners or a diagnosis of sexually transmitted infection (STI) within the year [16].

Many studies have examined changes in sexual behaviour among PrEP users and found a decrease in condom use since the initiation of PrEP and no change in the number of sexual partners [17, 18, 19]. Very few studies have so far investigated the impact of the diffusion of PrEP on the sexual behaviour and practices of people living with HIV. Hojilla et al. showed that bacterial STIs among men living with HIV who had sex with partners on PrEP were three times more prevalent than when partners were not on PrEP [20].

In France, health surveillance authorities have been alerting on the steady increase in the incidence of bacterial STIs since the early 2000s. This increase is particularly marked among MSM [4, 20–23] and predates the regular STI screening recommended for PrEP users, in a context of declining condom use in the general MSM population, including those living with HIV [21, 22, 24, 25]. Here, we are interested in the impact of PrEP diffusion. We hypothesized that (1) the diffusion of PrEP in the MSM population in France led to a change in the sexual behaviour of newly HIV‐positive MSM and (2) after acute HIV diagnosis, the reduced condom use and the diffusion of PrEP among their HIV‐negative partners could lead to an increased risk of bacterial STI acquisition. We, therefore, analysed data from the French ANRS PRIMO cohort to assess trends since 2014 in sexual behaviour and bacterial STIs among MSM living with HIV in the PrEP era, 6 months before seroconversion and in subsequent years. We also studied factors associated with inconsistent condom use (ICU) after HIV diagnosis, with reporting PrEP use by a sexual partner and with bacterial STI acquisition.

## 2 METHODS

### 2.1 Study design and population

The ongoing ANRS CO6 PRIMO cohort is a prospective, multicentre French cohort recruiting subjects during acute HIV‐1 diagnosis, whether symptomatic or not. Patients must be antiretroviral treatment naive, unless they have a history of PrEP or post‐exposure prophylaxis (PEP) use. Since 1996, 2516 patients have been included from 99 centres in France [10].

Participants were seen at enrolment at 1, 3, 6, 12 and then every 6 months until 2019, and annually since 2019. At each visit, a medical questionnaire including clinical, biological and therapeutic information is administered by a physician. In addition, self‐administered questionnaires on sexual behaviour are completed by the patient at baseline and annually. The baseline questionnaire seeks information on the protective measures used in the 6 months prior to HIV diagnosis. The follow‐up questionnaire provides information on the type (steady/occasional), number, and HIV status of sexual partners and condom use (always/often/rarely/never). Reasons for ICU (i.e. not always) have been recorded since 2013 and categorized into five non‐exclusive classes: TasP, use of PrEP by a partner, partner with the same HIV status, presumed low‐risk practices, other. The cohort was approved by the Paris Cochin ethics committee; ClinicalTrials.gov id: NCT03148964. All participants gave written consent.

MSM enrolled between 1 January 2014 and 31 December 2021, and who completed at least one self‐administered questionnaire were included in this analysis.

### 2.2 Statistical analyses

We studied trends in the number of sexual partners, ICU and reasons for ICU over time between 2014 and 2021 both before and after HIV diagnosis. The calendar changes in behaviour and practices were first studied graphically, stratified by the inclusion year to differentiate a period effect from a possible cohort effect, and then tested in random effects models.

The strength of the associations between the variables of interest and the three main outcomes (i.e. ICU, PrEP use by the partner, incident STI) was estimated using mixed generalized linear models. A logit link function was used and an exchangeable correlation structure was assumed. The variables of interest and those identified in the literature as potential confounders of the association studied were included in the multivariable analysis. Each MSM contributed to several annual visits.

We investigated the role of the following factors: the calendar period, age, education level, number of sexual partners over the last 12 months and some living conditions, such as professional activity (yes/no), being in a stable relationship, and tobacco and alcohol consumption, all these factors being able to be associated with sexual behaviour and STI acquisition. The size of the clinical centre (large above the 75th percentile vs. equal or below) was considered as a proxy for living in an urban area and also because the size of the centre can be associated with different medical practices. We pooled visit years by 2‐year periods, which also allowed us to individualize the 2020−2021 period, marked by the Sars‐Cov‐2 pandemic and during which important changes in sexual practices and the use of healthcare are likely to have occurred. The reference class was the 2016−2017 period, which corresponds to the introduction and diffusion of PrEP to the general MSM population in France. We categorized the number of partners into three classes: [0−1 partner], [2−10 partners] and [10 or more partners].

Factors associated with PrEP use by the sexual partner(s) were studied in the sub‐population of MSM reporting ICU in the previous 12 months, as information was only available in this case. Similarly, the role of PrEP use by the partner in the risk of STI was studied in this same sub‐population of MSM.

With regard to bacterial STI analyses, we considered infections with *N. gonorrhoeae* (NG), *C. trachomatis* (CT) and *T. pallidum* (Syphilis). Syphilis screening and, since 2015, CT and NG screening by PCR are recommended at HIV diagnosis in French guidelines [16]. Annual screening during follow‐up has also been recommended since 2015 for “all person living with HIV exposed to the risk of STIs.” In this study, a diagnosis of STI was defined by an evocative clinical manifestation, a specific antibiotic treatment or a biological result in favour of an acute infection at the visit or reported by the patient. A single diagnosis was considered for CT or NG polymerase chain reaction (PCR) positivity at one or several anatomical sites. For syphilis, in the absence of quantitative data available for the Veneral Disease Research Laboratory (VDRL) test, a new infection was defined by Treponema Pallidum Haemagglutination Assay (TPHA) and VDRL positivity more than 1 year after the last documented episode of syphilis. A prevalent STI was an STI diagnosed at the time of HIV diagnosis or in the previous month, whereas an incident STI was a new STI diagnosis during follow‐up. We defined a non‐at‐risk period following infection [26, 27]. The length of this period was decided on the basis of microbiological (natural history of each bacterial STIs), clinical and logistical considerations. These periods were of 7 days for NG, 3 weeks for CT and 1 year for syphilis. Taking this period into account as a risk‐free period enabled us to accurately assess the incidence of STIs, without overestimating it by wrongly counting two different diagnoses during two consecutive visits when it was the same episode. The non‐risk period following an STI diagnosis was subtracted from the total follow‐up time to estimate the incidence rate as the number of cases per 100 person‐years (PY) with its 95% confidence interval (CI). People entered the STI risk period from enrolment, unless they had a prevalent STI. In the latter case, they contributed person‐time at risk from the end of the non‐risk period. The time trends in the STI incidence rates were estimated and tested by negative binomial model [28].

Statistical analyses were performed using SAS version 9.4 software. All tests were two‐sided at a significance level of ∝ = 0.05. The database used was from 4 April 2022.

## 3 RESULTS

### 3.1 Population characteristics

**Figure 1:**
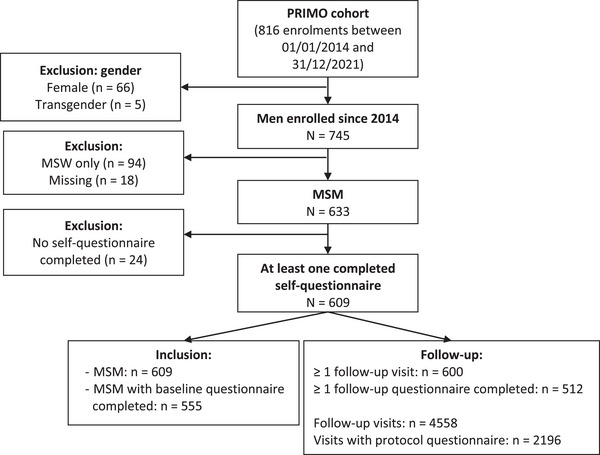
Flow chart.

Six hundred and nine MSM who were enrolled in the cohort between January 2014 and December 2021 and completed at least one self‐administered questionnaire were included in this study (Figure 1).

Most participants were born in France (92.8%), enrolled in a large clinical centre (77.5%) and had a university‐level education (62.3%), with no evolution of these distributions during the study period. The median age at HIV diagnosis was 35.5 years (IQR 27.4−44.9). The median follow‐up was 33.6 months (IQR: 12.0−49.0, range: 0−86.1) and the median number of completed self‐administered questionnaires during follow‐up was 3 (IQR: 2−5); the rate of completion was stable over time. Three participants died during follow‐up (0.5%) at 6.5, 17.1 and 33.1 months after enrolment.

### 3.2 Sexual behaviour before HIV diagnosis

**Figure 2:**
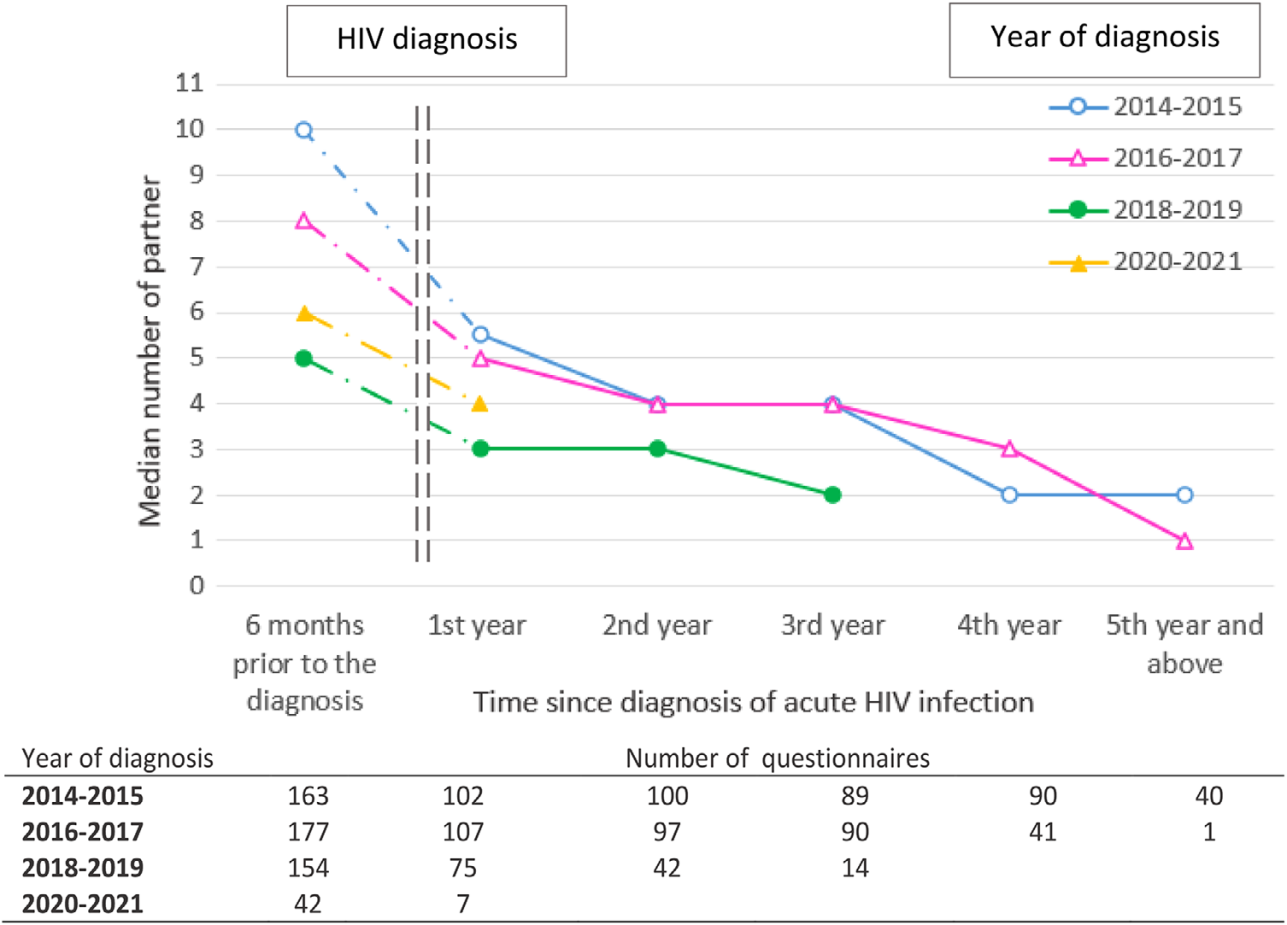
Change in the median number of partners within the 6 months prior to HIV diagnosis and during follow‐up by calendar period of diagnosis.

Almost all participants (99.3%) reported being sexually active in the 6 months prior to HIV diagnosis and the percentage was stable between 2014 and 2021. The proportion of men in a stable relationship tended to decrease over time (16.3% in 2014 vs. 6.4% in 2020−2021, *p* = 0.15) (Table 1). The median number of sexual partners in the 6 months prior to HIV diagnosis sharply decreased from 10 [IQR: 3−19] in 2014 to 4 [IQR: 2−10] in 2019 and 6 in 2020−2021 (Table 1 and Figure 2). Over the whole period 2014−2021, 85% of participants reported ICU during sex, with an increase over time up to 97.7% in 2020−2021 (*p* = 0.02). Overall, 7.2% (*n* = 40) reported having used PrEP at least once in the 6 months prior to HIV acquisition; they had a higher number of sexual partners than those who did not report PrEP use (median 12 [IQR: 6−20] vs. 7 [3−15], *p* = 0.05).

**Table 1:** Evolution of the characteristics and sexual behaviour in the 6 months preceding acute HIV diagnosis for 609 MSM enrolled in the ANRS PRIMO cohort

Year of enrolment	2014	2015	2016	2017	2018	2019	2020−2021	Total	*p*
**Number of MSM enrolled,** *N*	87	101	94	107	97	75	48	609	
**Born in France,** % (*n*)	96.6% (84)	91.1% (92)	91.5% (86)	92.5% (99)	90.7% (88)	92.0% (69)	97.9% (47)	92.8%^a^ (565)	0.55^*^
**University education level,** % (*n*)	61.3% (49)	67.7% (63)	60.2% (53)	68.9% (73)	57.3% (51)	58.6% (41)	63.0% (29)	62.3%^b^ (359)	0.52^*^
**In a relationship,** % (*n*)	26.4% (23)	27.7% (28)	27.7% (26)	15.9% (17)	18.6% (18)	26.7% (20)	18.6% (9)	23.2%^c^ (141)	0.15^*^
**In the 6 months preceding acute HIV diagnosis**									
**Number of male partners,** median (IQR)	10 (3−19)	9 (4−18)	6 (3−21)	8 (3−16)	5 (2−11)	4 (2−10)	6 (3−11)	7^d^ (3−15)	0.06^**^
**Number of subjects with >10 partners,** % (*n*)	45.7% (37)	40.2% (37)	38.8% (33)	38.8% (38)	25.3% (22)	21.7% (15)	27.3% (12)	34.9%^d^ (194)	0.001^*^
**Inconsistent condom use,** % (*n*)	77.8% (63)	88.0% (81)	80.0% (68)	82.7% (81)	88.5% (77)	85.5% (59)	97.7% (43)	85.5%^e^ (471)	0.02^*^
**Used PrEP at least once before diagnosis,** % (*n*)	6.9% (6)	5.0% (5)	4.3% (4)	3.7% (4)	8.2% (8)	13.3% (10)	6.3% (3)	7.2%^f^ (40)	0.91^*^

Abbreviations: IQR, interquartile range; MSM, men who have sex with men; PrEP, pre‐exposure prophylaxis.

Missing data: ^a^1.6%, ^b^6.1%, ^c^5.6%, ^d^3.6%, ^e^1.8%, ^f^24.5%.

* *p*‐trend; ^**^Jonckheere−Terpstra test.

### 3.3 Sexual behaviour after HIV diagnosis

**Figure 3:**
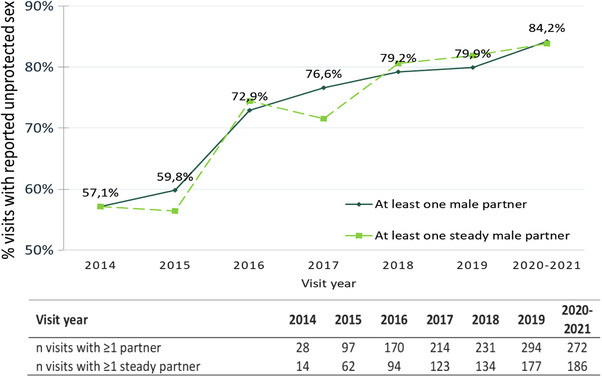
Percentage of visits during follow‐up in which inconsistent condom use was reported.

During follow‐up after HIV diagnosis, participants reported at least one sexual partner at a high and stable frequency (92.3% of visits), whereas their number of sexual partners decreased over time, whatever the year of diagnosis (Figure 2). In a sensitivity analysis including only MSM with both a self‐administered questionnaire at enrolment and at 1‐year, similar trends were observed. ICU increased from 57.1% of the visits in 2014 to 84.2% in 2020−2021, regardless of the type of partner, steady or occasional (Figure 3). In multivariable analysis, a more recent calendar period, an age of 30−35 years, having >10 partners were significantly associated with ICU (Table A1). Clinical centre size and other living conditions were not associated with ICU.

**Figure 4:**
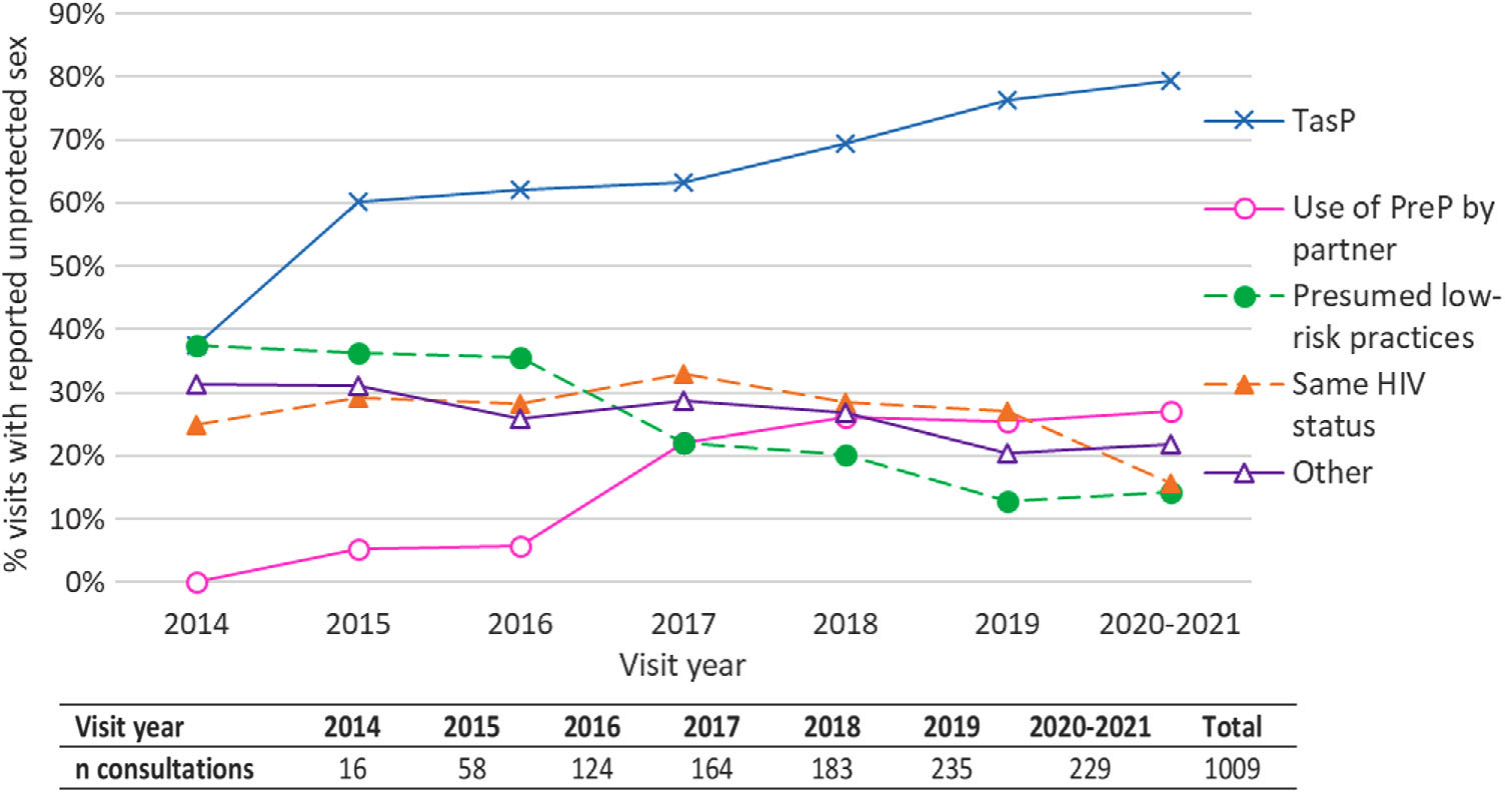
Changes over calendar time in the reasons for not using a condom during the follow‐up of HIV‐positive MSM reporting at least once inconsistent condom use in the previous 12 months.

Among the reasons cited for ICU, the TasP strategy increased over time, from 38% of visits in 2014 to 80% in 2020−2021, as did PrEP use by the partner(s), from 0% in 2014 to over 25% since 2018 (Figure 4), the two reasons, TasP and PrEP, being possibly associated, for example in 2019 in 20.4% of the visits with ICU. The use of PrEP by the partner as a reason for ICU was more frequent among MSM with a university level of education (24.5% vs. 14.3%, *p* = 0.002), not in a stable relationship (25.6% vs. 15.1%, *p* = 0.0004), with a higher number of partners (*p* < 0.0001) and being followed in a large clinical centre (23.6% vs. 14.3%, *p* = 0.01) (Table 2). In multivariable analysis, a more recent visit period, a higher level of education, a higher number of partners and not being in a stable relationship remained associated with more frequent reporting of PrEP use by a partner as a reason for ICU. Of note, age was not associated with reporting PrEP use by the partner(s).

**Table 2:** Factors associated with reporting PrEP use by at least one partner as a reason for inconsistent condom use among 424 MSM followed in the PRIMO cohort (*N* = 1009 visits)

				UNIVARIABLE			MULTIVARIABLE^a,^ ^*^		
	*N*	*n*	%	OR	95% CI	*p*	aOR	95% CI	*p*
**Visit period**									
2014‐2015	74	3	4.1	0.17	[0.04−0.68]	<0.0001	0.16	[0.04−0.67]	<0.0001
2016‐2017	288	43	14.9	1	−		1	−	
2018‐2019	418	108	25.8	2.4	[1.46−3.93]		3.44	[1.89−6.27]	
2020‐2021	229	62	27.1	2.7	[1.53−4.78]		4.17	[2.09−8.29]	
**Education level**									
Primary/secondary	308	44	14.3	1	−	0.002^b^	1	−	0.02
University	633	155	24.5	2.58	[1.44−4.63]		2.17	[1.14−4.10]	
**Professional activity**									
Unemployed	190	35	18.4	1	−	0.35			
Employed	819	181	22.1	1.29	[0.75−2.25]				
**Tobacco**									
No	553	126	22.8	1	−	0.49^c^			
Yes	450	88	19.6	0.86	[0.55−1.34]				
**Alcohol**									
No	216	50	23.2	1	−	0.49			
Occasional	580	122	21	0.74	[0.43−1.30]				
Regular	213	44	20.7	0.94	[0.48−1.83]				
**Clinical centre size**									
Other	238	34	14.3	1	−	0.01	1	−	0.41
Large	771	182	23.6	2.21	[1.20−4.07]		1.35	[0.66−2.75]	
**Age at visit, year**						0.62			
≤ 30	261	49	18.8	1	−				
[30−35]	171	37	21.6	1.1	[0.57−2.15]				
[35−45]	271	66	24.4	1.5	[0.80−2.81]				
≥ 45	306	64	20.9	1.33	[0.71−2.52]				
**Stable relationship**									
Yes	403	61	15.1	1	−	0.0004	1	−	0.02
No	606	155	25.6	2.36	[1.47−3.80]		1.95	[1.11−3.45]	
**Number of partners in the last 12 months**						<0.0001^d^			<0.0001
1	241	13	5.4	1	−		1	−	
[2‐10]	390	92	23.6	6.76	[3.32−13.76]		6.48	[2.87−14.63]	
[10 et +]	318	110	34.6	13.97	[6.57−29.72]		17.67	[7.17−43.55]	
**Total**	1009	216	21.4						

Abbreviations: aOR, adjusted OR; CI, confidence interval; OR, odds‐ratio.

Missing data: ^a^12.3%, ^b^6.7%, ^c^0.6%, ^d^5.9%.

* Model adjusted for all variables listed in the column.

### 3.4 Prevalence of bacterial STIs at acute HIV diagnosis and incidence during follow‐up

Screening of CT and NG infections at HIV diagnosis increased from 0% before 2015 to 59.6% in 2021 for the two infections, following the recommendations made in France in 2015. Syphilis screening was already high in 2014 (83.9% of HIV diagnoses) and remained so thereafter. In 2019, before the onset of the COVID‐19 pandemic, the prevalence of syphilis, gonorrhoea and chlamydia at HIV diagnosis was 8.0%, 14.7% and 18.7%, respectively; overall, 34.7% of MSM had a bacterial STI at acute HIV diagnosis.

**Figure 5:**
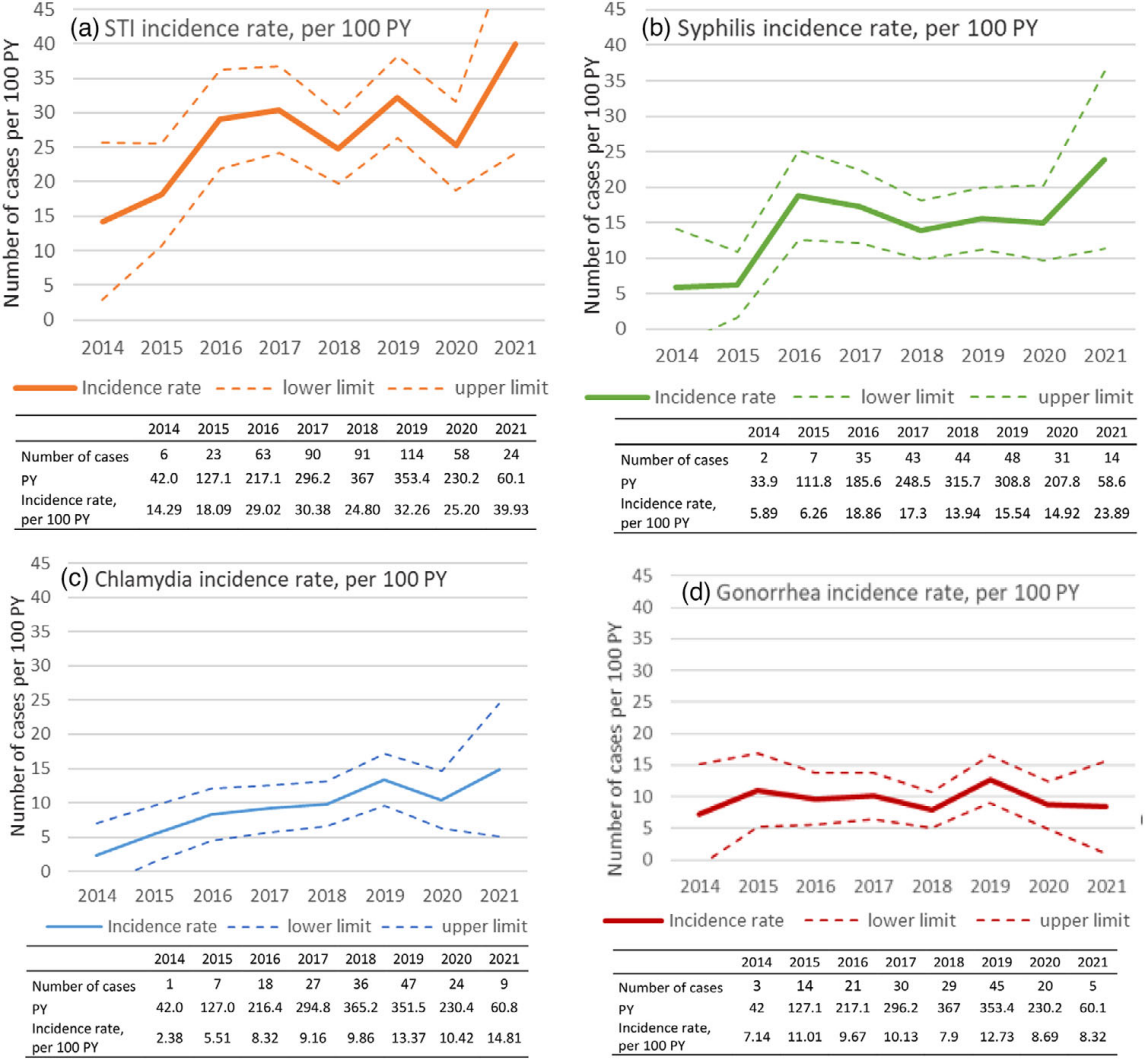
Change in the incidence rate of bacterial STIs by calendar period: (a) chlamydia and/or gonorrhoea and/or syphilis, (b) syphilis, (c) chlamydia and (d) gonorrhoea. Abbreviations: PY, person years; STI, sexually transmitted infection. 2021 Results must be interpreted with caution, as not all data are available.

During follow‐up, 260 participants (43.3%) had at least one new STI diagnosis, 169 incident CT, 167 NG and 224 syphilis infections. Among them, 122 individuals (20.3%) had STI diagnoses repeated over time. The incidence of STIs dramatically increased between 2014 and 2016 (corresponding to the implementation of the expert panel recommendations) and remained at a high and stable rate thereafter (*p* = 0.73) (Table A2). The STI incidence rate was 14 cases per 100 person‐years [95% CI: 3−26] in 2014 and 40 per 100 person‐years [24−56] in 2021 (Figure 5a). Each STI showed a distinct evolution. Concerning syphilis infections, the incidence rate significantly increased between 2014 and 2016, from 6 [0−14] to 19 cases [13−25] per 100 PY (*p* = 0.006), and then plateaued between 2016 and 2021 (*p* = 0.75) (Figure 5b). The incidence rate of chlamydia infections regularly increased by an average of 13% per year over the study period, from 2 [0−7] in 2014 to 15 cases [5−24] per 100 PY in 2021, *p* = 0.004 (Figure 5c). By contrast, the incidence rate of gonococcal infections was stable over time (*p* = 0.91) (Figure 5d). Caution must be exercised when interpreting results for 2021, as not all data are available.

Apart from the calendar period, risk factors for STI were ICU in the previous 12 months, not being in a stable relationship, a higher number of partners in the last 12 months and being currently aged < 35 years (Table A2 and Table 3). The use of PreP by the partner and the TasP strategy were associated with STIs in univariable analysis. In multivariable analysis, the risk of STIs was no longer significantly associated with the use of PrEP by the partner after adjustment for the calendar period and the number of partners. The risk of STIs tended to remain higher among subjects reporting no condom use due to the TasP method (*p* = 0.09), even after adjustment for the calendar period and the number of partners (Table 3).

**Table 3:** Factors associated with STI acquisition during follow‐up in the ANRS PRIMO cohort among 424 MSM living with HIV reporting inconsistent condom use in the past 12 months (*N* = 1009 visits)

				UNIVARIABLE			MULTIVARIABLE^a^, ^*^		
	*N*	*n*	%	OR	95% CI	*p*	aOR	95% CI	*p*
**Partner PrEP use**									
No	793	199	25.1	1	−	0.009	1	−	0.6
Yes	216	74	34.3	1.68	[1.14−2.48]		1.12	[0.74−1.68]	
**TasP**									
No	299	68	22.7	1	−	0.08	1	−	0.09
Yes	710	205	28.9	1.39	[0.96−2.01]		1.41	[0.94−2.10]	
**Visit period**						0.01			0.01
2014‐2015	74	11	14.9	0.36	[0.16−0.81]		0.38	[0.17−0.88]	
2016‐2017	288	77	26.7	1	−		1	−	
2018‐2019	418	126	30.1	1.29	[0.87−1.90]		1.43	[0.95−2.16]	
2020‐2021	229	59	25.8	0.97	[0.61−1.54]		1.08	[0.67−1.76]	
**Age at visit, year**						0.09			0.09
≤ 30	261	78	29.9	1			1		
[30−35]	171	58	33.9	1.19	[0.72−1.97]		1.18	[0.69−2.02]	
[35−45]	271	60	22.1	0.64	[0.39−1.04]		0.62	[0.37−1.05]	
≥ 45	306	77	25.2	0.75	[0.47−1.21]		0.74	[0.45−1.22]	
**Number of partners in the past 12 months**						<0.001^a^			0.009
1	241	40	16.6	1	−		1	−	
[2‐10]	390	114	29.2	2.13	[1.36−3.34]		1.78	[1.09−2.90]	
[10 et +[	318	107	33.7	2.66	[1.66−4.27]		2.28	[1.34−3.88]	
**Total**	1009	273	27.1						

Abbreviations: aOR, adjusted OR; CI, confidence interval; MSM, men who have sex with men; OR, odds‐ratio; PrEP, pre‐exposure prophylaxis; TasP, treatment as prevention.

^a^ 5.9% missing data.

* Adjusted for calendar period of the visit, current age, lifestyle, number of partners in the past 12 months, PrEP and TasP use.

## 4 DISCUSSION

This study was conducted in the context in which the diffusion of the TasP strategy and, more recently, PrEP have modified the epidemiology of HIV and STIs, particularly among MSM.

We had hypothesized that the diffusion of PrEP among MSM would lead to a change in the characteristics of newly diagnosed HIV‐positive MSM. Indeed, we observed a decrease between 2014 and 2021 in the number of sexual partners in MSM with HIV just before their diagnosis. This is most likely due to a (positive) selection effect, caused by the diffusion of effective PrEP among MSM with multiple partners or who had contracted an STI within the year. In other words, a proportion of this key population who had benefited from a PrEP prescription since 2015−2016 was no longer at risk of HIV acquisition because of the strong protection afforded by PrEP, and was thus not included in the cohort. In support of this interpretation, MSM who had used PrEP at least once prior to HIV diagnosis had a higher number of partners than non‐PrEP users. Therefore, subjects living with HIV included in the ANRS PRIMO cohort in recent years are those who did not have access to, did not want, did not meet the indications, in particular in terms of the number of partners, or struggled with adherence. These results clearly argue for an expansion of the indications for PrEP. Data are still lacking on the impact of the implementation of PrEP on the incidence of HIV in MSM in France; it is of interest, however, to note the decline in the number of enrolments in the cohort, which began before the Covid pandemics onset.

The number of sexual partners also tended to decrease during follow‐up after HIV diagnosis. This has not been described for MSM with HIV thus far. The stable completion rate of the questionnaires over the study period and the changes in condom use practices do not argue in favour of increased under‐reporting of the number of sexual partners. Molina et al. showed the same evolution for HIV‐negative MSM using PrEP over time [29].

Our results are consistent with those in the literature concerning the increase in condomless sex among MSM in recent years [11, 17, 18, 24, 25]. The TasP strategy was the main reason for not using condom, up to 80% in the most recent years. ICU has been increasingly motivated by PrEP use by the partner(s), reported in more than 25% of visits since 2018, and was frequently associated with the TasP strategy and a higher level of education. This association could be partially due to a social homogamy effect in the choice of partners in the context of better access to care and prevention by the more highly educated [30, 31]. Participants who reported PrEP use by partner also reported a higher number of partners: beyond the purely statistical aspect of the association (the probability of having at least one partner using PrEP increasing with the number of partners), this may reflect the socio‐sexual network of MSM with a large number of sexual partners.

The increase over time in the prevalence of STIs at the time of acute HIV diagnosis is consistent with the increase reported by the French health surveillance agency in the general MSM population [3, 4]. However, after HIV diagnosis, the incidence of STIs has been stable over time since 2016. Several reasons may explain this apparent discordance. We may have underestimated STIs incidence during follow‐up, partially related to insufficient screening coverage during follow‐up. For example, the incidence of STIs in the ANRS Optiprim2 trial, in which participants were monitored more closely from the time of seroconversion diagnosis, was 70 per 100 person‐years [32]. Bacterial STIs may also be underdiagnosed because of the current national recommendations, which only target persons living with HIV exposed to the risk of STIs [33], knowing that it is not always possible to determine whether the patient is exposed to the risk of STIs. On the other hand, the non‐increase in the incidence of STIs during follow‐up may be real, as the number of sexual partners decreased over time after HIV diagnosis.

Subjects under 35 years of age, not in a stable relationship and with a higher number of partners were those with the highest risk of STI. Reporting having a partner using PrEP as a reason for ICU was associated with an increased risk of STIs. These results are similar to those published by Hojilla et al. in 2020 in a U.S. cohort of MSM with HIV [20]. In our study, the association between partner PrEP use and STI acquisition was related to an association with the number of sexual partners and the calendar period, and was no longer observed after adjustment for these two factors. In any case, STI screening should be done, even for people with a single partner, and prevention methods, such as the use of doxycycline post‐exposition, should be encouraged [34, 35].

Among the main strengths of our study were longitudinal data collection in a large sample of MSM followed in the ANRS PRIMO cohort from seroconversion diagnosis, using self‐administered questionnaires covering the 6‐month period before and after HIV diagnosis. This allowed us to report on the evolution of sexual practices over several years. The questionnaire completion rate during follow‐up was stable over time. Self‐administered questionnaires minimize under‐reporting of sensitive data, such as sexual behaviour, compared to questionnaires administered face‐to‐face by the physician, as was observed in the early years of the cohort [10]. Mostly, we previously showed that this cohort was able to capture changes in sexual practices related to a TasP effect [10, 11].

Among the limitations, PrEP use by a sexual partner may have been underestimated, as the cohort participant may have not been aware of such use, and information was only requested when subjects reported ICU. Therefore, we could not assess the mediating role of ICU in the association between the use of PrEP by a partner and acquiring an STI. However, we could assess the direct impact of PrEP use by a partner in the acquisition of an STI among participants reporting condomless sex, the most numerous ones. Concerning PrEP use prior to HIV diagnosis, information was limited to the 6 months prior to HIV diagnosis; lifetime PrEP use data should be collected in the future, as well as on knowledge about PrEP and reasons for non‐use. Finally, information on certain STI risk factors described in the literature is absent. The PRIMO cohort, although rich in information, does not yet provide information on the characteristics of the sexual network, the use of recreational drugs during sex or the concomitance and rate of exchange of partners, sex groups, or places where partners go out or meet [27, 36].

## 5 CONCLUSIONS

We show that ICU is increasingly common among MSM living with HIV and that the use of PrEP in their sexual networks has also increased. The diffusion of PrEP in the overall general MSM population has led to a change in the characteristics of newly HIV‐diagnosed individuals in the cohort, with the most exposed individuals now acquiring HIV less frequently due to PrEP. This clearly raises the question of expanding the indications for PrEP in France, expanding access and promoting adherence [37]. The incidence of bacterial STIs remains high in MSM. This also shows the need for continued improvements in screening, prevention and treatment. More regular STI screening is needed, including regular at‐home STI screening [38]. Prevention methods must be improved, through antibiotic pre‐ or post‐exposition or vaccination [34, 35].

## COMPETING INTERESTS

The authors have no competing interests.

## AUTHORS’ CONTRIBUTIONS

VB and LM contributed to the study design. CG, AE, KC, PDB, LL and RS contributed to the data preparation. VB, KC, PDB and LL performed the statistical analysis. VB and LM wrote the first version of the article. All authors contributed to the interpretation of the results and critically revised the manuscript.

## FUNDING

The ANRS PRIMO cohort study is funded by ANRS‐MIE (French national agency for research on AIDS and viral hepatitis‐Emerging Infectious Diseases), Paris. KC received a post‐doctoral scholarship from Sidaction to initiate the work.

## Data Availability

Data are available on request due to privacy/ethical restrictions.

## 1

**Table A1:** Factors associated with inconsistent condom use in the previous 12 months among subjects reporting at least one male partner over the same period (*n* = 1351 visits among 492 MSM)

				UNIVARIABLE			MULTIVARIABLE^a^,*		
	*N*	*n*	%	OR	95% CI	*p*	aOR	95% CI	*p*
**Visit period**									
2014‐2015	125	4	59.2	0.29	[0.15−0.54]	< 0.0001^b^	0.33	[0.17−0.65]	< 0.0001
2016‐2017	384	288	75	1	−		1	−	
2018‐2019	525	418	79.6	1.75	[1.13−2.73]		2.03	[1.28−3.24]	
2020‐2021	272	229	84.2	3.19	[1.77−5.74]		4.23	[2.27−7.87]	
**Education level**									0.28
Primary/secondary	414	308	74.4	1	−	0.13^c^	1	−	
University	804	633	78.7	1.49	[0.89−2.48]		1.36	[0.78−2.37]	
**Professional activity**									
Unemployed	242	188	78.5	1	−	0.72^b^			
Employed	1064	819	77	0.91	[0.55−1.51]				
**Tobacco**									
No	716	553	77.2	1	−	0.84^d^			
Yes	583	450	77.2	0.96	[0.63−1.46]				
**Alcohol**									
No	286	216	75.5	1	−	0.22			
Occasional	764	580	75.9	1.21	[0.74−1.98]				
Regular	255	213	83.5	1.77	[0.92−3.40]				
**Clinical centre size**									
Other	304	238	78.3	1	−	0.94^b^			
Large	1002	771	76.9	0.98	[0.54−1.74]				
**Age at visit, year**						0.1			0.28
≤ 30	353	261	73.9	1	−		1	−	
[30−35]	207	171	82.6	2.34	[1.19−4.60]		2.14	[1.00−4.62]	
[35−45]	348	271	77.9	1.47	[0.80−2.69]		1.27	[0.65−2.46]	
≥ 45	398	306	76.9	1.56	[0.85−2.87]		1.25	[0.64−2.44]	
**Stable relationship**									
Yes	510	403	79	1	−	0.31			
No	796	606	76.1	0.8	[0.53−1.23]				
**Number of partners in the last 12 months**						0.0002^d^			<0.0001
1	332	241	72.6	1	−		1	−	
[2‐10]	548	390	71.2	1.04	[0.67−1.61]		1.1	[0.68−1.78]	
[10 et +]	365	318	87.1	2.84	[1.62−5.00]		3.72	[1.93−7.17]	
**Total**	1351	1009	74.7						

Abbreviations: aOR, adjusted OR; CI, confidence interval; MSM, men who have sex with men; OR, odds‐ratio; PrEP, pre‐exposure prophylaxis; TasP, treatment as prevention.

Missing data: ^a^11.1%, ^b^3.3%, ^c^10.1%, ^d^3.8%.

* Model adjusted for all variables listed in the column.

**Table A2:** Factors associated with the acquisition of an STI during follow‐up in the ANRS PRIMO cohort among 492 HIV‐positive MSM reporting at least one male partner in the previous 12 months (*N* = 1351 visits)

				UNIVARIABLE			MULTIVARIABLE^a,^ *		
	*N*	*n*	%	OR	95% CI	*p*	aOR	95% CI	*p*
**Consistent condom use**									
Yes	297	43	14.5	1	−	<0.0001^b^	1	−	0.0006
No	1009	273	27.1	2.42	[1.60−3.64]		2.13	[1.39−3.27]	
**Visit period**						0.0002			0.0004
2014‐2015	128	13	10.2	0.27	[0.13−0.53]		0.3	[0.14−0.63]	
2016‐2017	399	96	24.1	1	−		1	−	
2018‐2019	545	146	26.8	1.28	[0.90−1.81]		1.44	[0.99−2.08]	
2020‐2021	279	69	24.7	1.09	[0.71−1.65]		1.15	[0.74−1.93]	
**Education level**									
Primary/secondary	430	107	24.9	1	−	0.6			
University	832	196	23.6	0.91	[0.62−1.32]				
**Professional activity**									
Unemployed	247	61	24.7	1	−	0.84			
Employed	1104	263	23.8	0.96	[0.65−1.43]				
**Tobacco**									
No	740	179	24.2	1	−	0.94^c^			
Yes	604	144	23.8	1.01	[0.73−1.40]				
**Alcohol**									
No	294	71	24.2	1	−	0.81			
Occasional	787	184	23.4	0.91	[0.61−1.35]				
Regular	269	69	25.7	1.02	[0.62−1.66]				
**Clinical centre size**									
Other	316	74	23.4	1	−	0.94			
Large	1035	250	24.2	1.05	[0.68−1.63]				
**Age at visit, years**						0.07			0.04
≤ 30	359	97	27	1	−		1	−	
[30−35]	215	64	29.8	1.21	[0.76−1.92]		1.17	[0.71−1.93]	
[35−45]	360	72	20	0.68	[0.43−1.06]		0.62	[0.39−1.01]	
≥ 45	417	91	21.8	0.75	[0.49−1.16]		0.68	[0.43−1.09]	
**Stable relationship**									0.002
Yes	529	95	18	1	−	< 0.0001	1	−	
No	822	229	27.9	1.96	[1.40−2.75]		1.78	[1.20−2.59]	
**Number of partners declared at the visit**						0.003^d^			0.05
1	334	57	17.1	1	−		1	−	
[2‐10]	555	139	25	1.64	[1.10−2.44]		1.43	[0.94−2.19]	
[10 et +]	367	111	30.2	2.14	[1.38−3.31]		1.81	[1.13−2.91]	
**Total**	1351	324	24						

Abbreviations: aOR, adjusted OR; CI, confidence interval; MSM, men who have sex with men; OR, odds‐ratio; PrEP, pre‐exposure prophylaxis; TasP, treatment as prevention.

Missing data: ^a^7.8%, ^b^3.3%, ^c^0.5%, ^d^7%.

* Model adjusted for all variables listed in the column.
